# Small molecule mediators of host-*T*. *cruzi-*environment interactions in Chagas disease

**DOI:** 10.1371/journal.ppat.1012012

**Published:** 2024-03-08

**Authors:** Godwin Kwakye-Nuako, Caitlyn E. Middleton, Laura-Isobel McCall

**Affiliations:** 1 Department of Chemistry and Biochemistry, University of Oklahoma, Norman, Oklahoma, United States of America; 2 Department of Biomedical Sciences, School of Allied Health Sciences, College of Health and Allied Sciences, University of Cape Coast, Cape Coast, Ghana; 3 Department of Chemistry and Biochemistry, San Diego State University, San Diego, California, United States of America; University of Bern: Universitat Bern, SWITZERLAND

## Abstract

Small molecules (less than 1,500 Da) include major biological signals that mediate host-pathogen-microbiome communication. They also include key intermediates of metabolism and critical cellular building blocks. Pathogens present with unique nutritional needs that restrict pathogen colonization or promote tissue damage. In parallel, parts of host metabolism are responsive to immune signaling and regulated by immune cascades. These interactions can trigger both adaptive and maladaptive metabolic changes in the host, with microbiome-derived signals also contributing to disease progression. In turn, targeting pathogen metabolic needs or maladaptive host metabolic changes is an important strategy to develop new treatments for infectious diseases. *Trypanosoma cruzi* is a single-celled eukaryotic pathogen and the causative agent of Chagas disease, a neglected tropical disease associated with cardiac and intestinal dysfunction. Here, we discuss the role of small molecules during *T*. *cruzi* infection in its vector and in the mammalian host. We integrate these findings to build a theoretical interpretation of how maladaptive metabolic changes drive Chagas disease and extrapolate on how these findings can guide drug development.

## 1. Introduction

### 1.1. Small molecules, metabolites, and metabolomics

**Fig 1:**
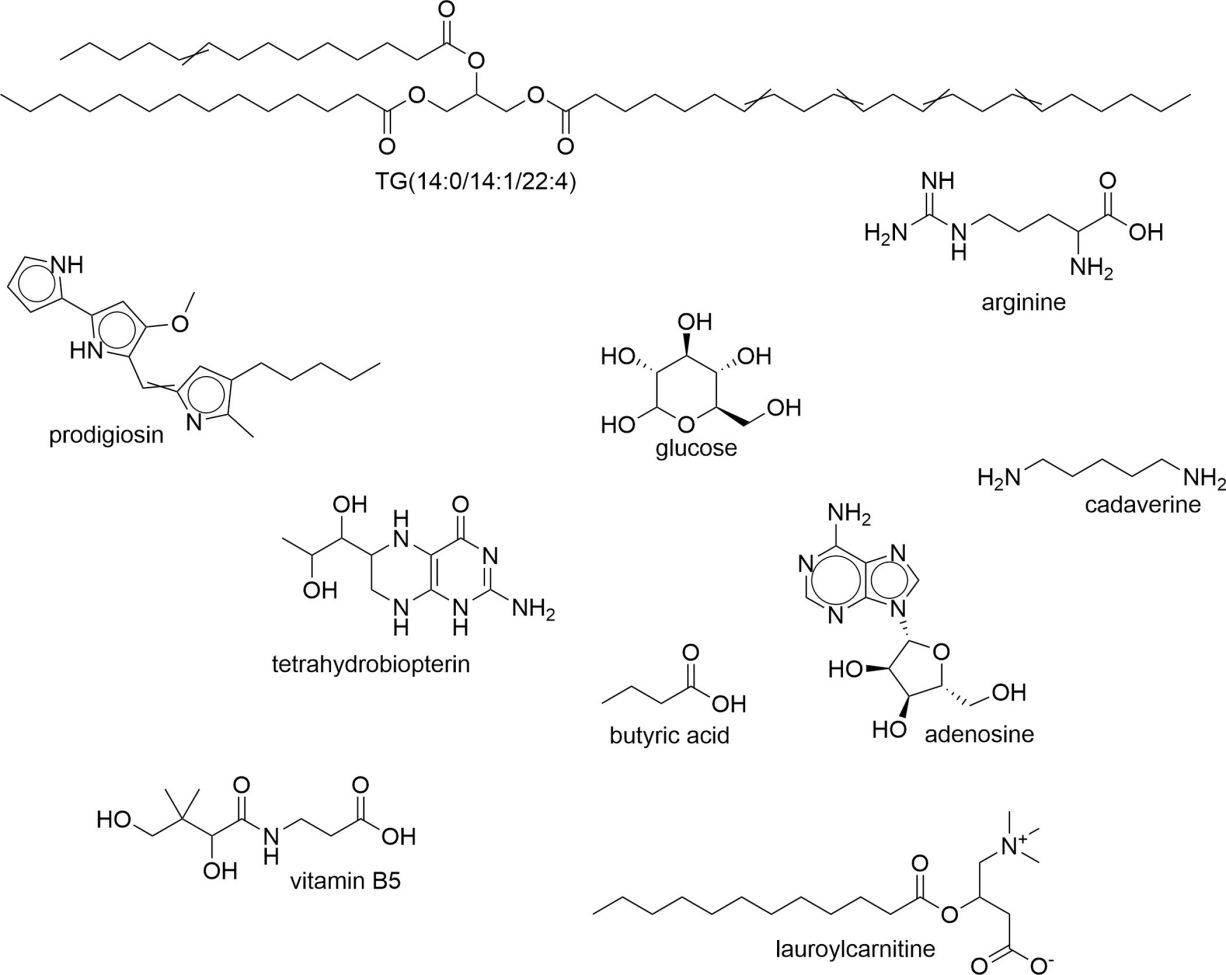
The structural diversity of metabolites: Representative metabolite structures, as discussed in the text. The depicted molecules span a range of chemical classes, including amino acids (arginine), nucleosides (adenosine), coenzymes (tetrahydrobiopterin), and fatty acids (TG). Figure created in ChemDraw 20.1.

Metabolites are small organic molecules (50 to 1,500 Da) that play significant roles as the intermediate or end product of metabolic activities [1]. They are categorized into primary and secondary metabolites [2]. Primary metabolites are those directly involved in biological mechanisms such as growth, development, and reproduction, for example, amino acids. Their chemical transformations in metabolism are functionally described in cellular biochemistry [3]. Consequently, primary metabolism and central carbon metabolism are often used interchangeably. Secondary metabolites are not directly involved in the metabolism of the biological system but rather can mediate activities that enhance its survival [4]. In addition to the primary and secondary metabolites, some compounds of drug and food origin are also small molecules that can affect host-pathogen interactions [5]. Here, we will use “metabolites” in the broadest sense encompassing all those molecules, including lipids (**Fig 1**) [6].

Metabolomics is the quantitative or semiquantitative analysis of a broad range of metabolites [7,8]. The chemicals of interest in each investigation determine the selected metabolomics workflow. In a targeted metabolomics experiment, only metabolites from a preexisting list are analyzed, often from particular pathways [9]. The untargeted approach, on the other hand, identifies as many metabolites as possible in a biological sample, without a preexisting inclusion list and thus minimizing prior bias. Thus, the number of metabolites analyzed is usually greater in untargeted workflows than in targeted workflows [3,9]. Mass spectrometry is often employed in metabolomics to detect, identify, and quantify metabolites, commonly in combination with liquid chromatography for the characterization of polar or semipolar metabolites, or gas chromatography for less polar or small volatile metabolites, or for polar molecules after derivatization. An alternative technique is nuclear magnetic resonance [8].

The functions associated with these small molecules include structure, signaling, catabolic activities, defense mechanisms, etc. [10]. For instance, some metabolites play a significant role in interspecies crosstalk [8]. In the context of Chagas disease (CD), metabolites play a key role in parasite-vector interactions, parasite-mammalian host interactions, and parasite-microbiome interactions [11]. Exogenous metabolites, such as those derived from the diet, can also influence disease progression. These pathways have been exploited for drug development. In this review, we cover recent research on these topics, as well as current challenges in the field of CD-metabolite interactions.

### 1.2. *T*. *cruzi* infection and Chagas disease

*Trypanosoma cruzi* is the parasite responsible for CD, localized mostly in Latin America and the Southern United States of America, with sporadic cases worldwide due to population movements. This parasite infects 5 to 6 million people in endemic areas [12], resulting in approximately 12,000 deaths annually with 70 million people at risk of infection [13]. In the mammalian host, CD symptoms are localized in the gastrointestinal tract and heart, with symptoms of cardiac arrhythmias, cardiomyopathy, apical cardiac aneurysms, megacolon, and megaesophagus [14]. The parasite itself has broader tropism, most frequently including the gastrointestinal tract, in a host- and parasite strain-specific manner [15].

The forms of the parasite that exist in the mammalian host are the intracellular amastigotes and the extracellular trypomastigotes. Triatomine insects are the proven vectors of *T*. *cruzi*. They depend on vertebrate hosts for blood meals, during which they take up trypomastigotes and amastigotes. In the triatomine stomach, the trypomastigotes then transform into epimastigotes. Epimastigotes make their way into the triatomine midgut to multiply. In the triatomine hindgut, epimastigotes differentiate into infective metacyclic trypomastigotes. These trypomastigotes get released with triatomine feces and urine during or after a blood meal, enabling transmission to the vertebrate host to complete the *T*. *cruzi* lifecycle [16].

## 2. The triatomine vector and metabolites: Four-way interactions between insect, insect microbiome, parasite, and mammalian host

Most research on *T*. *cruzi* interactions with triatomines has focused on proteins and on reactive oxygen and nitrogen species [17]. However, *T*. *cruzi* in the triatomine gut will also encounter both insect-derived and triatomine gut microbiome-derived metabolites. For instance, prodigiosin (**Fig 1**), produced by *Serratia marcescens* in the *Rhodnius prolixus* microbiota, can kill *T*. *cruzi* [18,19]. Conversely, the vector microbiome can synthesize all 8 group B vitamins and tetrahydrofolate, which may provide essential nutrients for *T*. *cruzi* given its auxotrophies (see below) [20,21]. *T*. *cruzi* is also an auxotroph for many amino acids [22] and can use amino acids as energy sources and inducers of parasite differentiation [23–25]. *Actinomyces* in the vector microbiome may be a source of histidine [26], and functional capabilities across amino acid metabolic pathways were found by metagenomic analysis of the *R*. *prolixus* gut microbiome [21]. Additional relevant metagenome-supported metabolic capabilities include carbohydrate and nucleotide metabolism [21], which may likewise help address *T*. *cruzi* auxotrophies and energy needs. Indeed, triatomine guts and feces contain a diverse range of chemical classes, with lipids the most abundant [11,27]. Free fatty acids are an important energy source for *T*. *cruzi* epimastigotes, if glucose is limiting, and are sufficient to induce *T*. *cruzi* development from epimastigotes to metacyclic trypomastigotes [28–30]. Purines and pyrimidines are present and may serve as sources for parasite proliferation. Interestingly, fecal nucleotide levels differed between triatomine species, possibly leading to differential restrictions on parasite growth [27].

This fecal material will be transferred through the bite wound into the mammalian host along with salivary metabolites and the parasite [16]. The salivary biomolecules of most hematophagous disease-transmitting vectors have been explored over the years to understand their role in infection. However, they have been studied in triatomines primarily at the protein level, with limited small molecule studies [31,32], except for lysophosphatidylcholine. Lysophosphatidylcholine is found in the saliva of *R*. *prolixus* [33]. This compound prevents blood clotting [33], and by inference is important for the feeding behavior of the vector. Additionally, lysophosphatidylcholine inhibits antiparasitic immune responses, and preadministration prior to *T*. *cruzi* infection increased parasitemia [34,35]. *T*. *cruzi* itself is also a source of lysophosphatidylcholine [36,37]. An intriguing possibility, given the role of host lysophosphatidylcholine in *Plasmodium* sexual differentiation [38], is that triatomine salivary levels of this molecule may also be affecting parasite differentiation and virulence. Sandfly salivary adenosine (**Fig 1**) can reshape antiparasitic immune responses to prevent killing of the related parasite *Leishmania* and help *Leishmania* establish itself [39]. A similar mechanism may occur for adenosine from triatomines, either fecal or salivary. Given that the nutritional environment influences parasite infectivity [40], these results provide impetus to further explore the range of metabolites involved in reshaping the microenvironment as *T*. *cruzi* transitions from vector to host.

## 3. Importance of exogenous metabolites for *T*. *cruzi* during mammalian infection

As described above, parasite auxotrophies may play a role in vector colonization by *T*. *cruzi* and will likewise be important during mammalian infection. Specifically, *T*. *cruzi* is reported to be unable to synthesize purines, heme, folate, biopterin (important as a precursor for the enzyme cofactor tetrahydrobiopterin (**Fig 1**)) [22,41], the diamines putrescine and cadaverine (**Fig 1,** important precursors for the antioxidant trypanothione [42]), vitamin B1, vitamin B3, vitamin B5 (**Fig 1**), vitamin B6, vitamin B7, vitamin B12 [43], and 9 amino acids (isoleucine, leucine, valine, tryptophan, phenylalanine, tyrosine, lysine, histidine, and arginine (**Fig 1**)) [22,44,45]. These nutrients are available to *T*. *cruzi* in the mammalian cytosol [46–49], though levels of tetrahydrobiopterin may become limiting under inflammatory conditions [50]. This inflammation-induced restriction is counterbalanced by the observed increase in the expression of host genes involved in tetrahydrobiopterin production in infected cells in vitro [51], which could be induced by the parasite to favor its growth, or as a response from the host to compensate for biopterin depletion by the parasite. Regardless of the cause, this increase benefits *T*. *cruzi*, as silencing of host enzymes involved in tetrahydrobiopterin synthesis inhibits in vitro *T*. *cruzi* growth [52].

However, several of these auxotrophies were defined by extension from the better-studied trypanosomatids *Leishmania* and *T*. *brucei*. Improved genomic studies, and *T*. *cruzi-*specific studies, may in some cases lead to redefining of these restrictions [53], with the caveat that presence of a gene in the parasite genome does not necessarily mean its expression across stages and across sites of infection. Furthermore, when performed in *T*. *cruzi*, functional assays often used exclusively epimastigotes in culture media, which may not fully represent the in vivo environment encountered by amastigotes or trypomastigotes, where combinations of nutrients may also be restricted or where nutrient levels may fluctuate in response to inflammation [54]. In particular, even though *T*. *cruzi* has pyrimidine biosynthetic capabilities, inhibiting host pyrimidine nucleoside production by RNAi restricts *T*. *cruzi* growth during in vitro epithelial cell infection [52], indicating that genetically defined auxotrophies do not account for all parasite nutritional restrictions in vivo.

Indeed, beyond the restrictions of auxotrophy, *T*. *cruzi* benefits from importing nutrients from the host that favor its growth or metabolic activities. Parasite metabolic activities, and thus scavenging needs, vary between stages of parasite invasion. For example, parasite glycolytic transcripts decrease for the first 4 h of intracellular infection, followed by a rebound [55]. *T*. *cruzi* in vitro intracellular amastigote proliferation rate is dependent on glucose (**Fig 1**) and glutamine availability and on glycolysis [56,57]. Infected cardiomyocyte and fibroblast cultures present with comparable increases in glucose consumption from the culture media, increased glycolytic gene expression, and increased lactate secretion [57,58]. Given that inhibiting glucose import had stronger effects than targeted inhibitors of glycolysis [57], that decreased levels of lactate dehydrogenase A (LDHA), the enzyme catalyzing interconversion of pyruvate and lactate and a promoter of host glycolysis, increased intracellular parasite levels [52], and that *T*. *cruzi* can use host-derived glucose [58], these observations suggest that the observed increase in glucose import may directly be fueling *T*. *cruzi* metabolism. However, it should be noted that amastigote glucose import capabilities may be context and time point dependent [59].

Host lipid biosynthetic genes are increased by 48 to 72 h postinfection [51], with accumulation of lipid bodies in infected HeLa epithelial cells [60] and in heart tissue [61]. Cells deficient in several lipid biosynthesis genes showed reduction in parasite burden [52]. *T*. *cruzi* gene expression and protein expression patterns based on in vitro cultures indicate a shift in parasite energy usage toward amino acid and fatty acid oxidation [51,62]. Parasite fatty acid oxidation transcripts increase steadily up to a plateau at 24 h postinfection, though the timing of these changes is strain dependent [55]. Furthermore, while able to produce major lipids, *T*. *cruzi* in vitro intracellular amastigotes scavenge most of their triacylglycerols and diacylglycerols from the host and use host lipids as sources for long-chain glycerophosphocholines [63]. Consequently, the *T*. *cruzi* amastigote lipidome is dependent on the host cell from which they are derived [63]. Jointly, these characteristics would cause *T*. *cruzi* to benefit from the observed increased lipid storage, either by providing substrates for parasite β-oxidation, or precursors for parasite membrane lipids. *T*. *cruzi* grew better under in vitro conditions that favor β-oxidation over glucose oxidation in epithelial cells [52]. This may also be through scavenging of host β-oxidation intermediates that then fuel *T*. *cruzi* β-oxidation, or from the energy and reducing intermediates produced by this pathway in the host. Host β-oxidation may also help promote host cell survival to give *T*. *cruzi* the time to develop, whereas premature host cell mortality would prevent *T*. *cruzi* expansion.

These observations with regard to parasite fatty acid oxidation are not necessarily contradictory with the observation of parasite glucose usage, since fatty acid and glucose oxidation can coexist to meet cellular energy needs and the flexibility to use both pathways for energy generation may help facilitate the broad parasite tropism observed during acute infection in vivo [64]. Given that transcript levels do not necessarily reflect protein levels, and neither measurement reflects metabolic enzyme activity, which may be regulated allosterically, by covalent modifications, by protein–protein interactions, or by substrate and product availability, methods that directly quantify nutrient usage by the parasite are overall more reliable, though more challenging to implement. Flux-based studies are the most rigorous and least confounded of these approaches. Inhibitor studies can provide valuable insight but should also be considered with caution, as some compounds can promiscuously inhibit multiple pathways. Even a compound targeting one metabolic pathway with high selectivity may unintentionally cause effects on other metabolic pathways as cells seek to compensate for inhibition by up-regulating other pathways. A further challenge with all these studies is that none of them directly measured parasite metabolism in situ, within tissues. Improvements in the spatial resolution of imaging mass spectrometry and single-cell metabolomics may help partially address these concerns [65,66].

A preference for host cells that rely on β-oxidation has been interpreted to explain parasite tropism to organs like the heart [52,67], likely due to the parasite growth-promoting mechanisms hypothesized above. However, *T*. *cruzi* has strong tropism to gastrointestinal smooth muscle [15], which prefers to use glucose to fuel contractions [68]. Furthermore, the β-oxidation inhibitor etomoxir did not reduce intracellular amastigote parasite burden in epithelial cells in vitro, suggesting that host β-oxidation may be less critical for *T*. *cruzi* growth in this context [69]. These results should, however, be interpreted with care, given that parasite substrate utilization in tissues may differ from what was defined *in vitro*. Preexisting gradients of metabolite availability may also shape *T*. *cruzi* tropism [70,71]. For example, higher AMP at the heart apex could be restricting *T*. *cruzi* through activation of AMP-activated protein kinase (AMPK) [52,71]. However, modulating several individual metabolic pathways has yet to change parasite spatial distribution ([70] and our own observations), suggesting that altering *T*. *cruzi* spatial distribution would require combinations of metabolic effects, rather than individual signals.

## 4. Host metabolic changes: Protective or maladaptive?

As described above, some of the infection-induced changes in host metabolic pathways may be beneficial to the parasite, by providing nutrients that fuel parasite metabolism. In contrast, restricting availability of metabolites essential to *T*. *cruzi* could restrict parasite growth. Indeed, reduced levels of purines have been observed in mouse models and in human systems [72–74], along with many other nutrients essential to *T*. *cruzi*: vitamins B2, B6, and B12, lysine, valine, arginine, and phenylalanine [74], though with some conflicting results between studies [72]. A caveat is that reduced levels of these nutrients cannot automatically be interpreted as direct uptake by the parasite, given the low parasite burden during chronic infection and the observation that some of these changes persist even after antiparasitic treatment, such as partial purine depletion [73,75].

Changes in host metabolism may alternatively contribute to exacerbating disease symptoms. Indeed, sites of persistent or worsening metabolic alterations during experimental CD are distal to sites of highest parasite burden but concur with sites of CD symptoms (heart apex, esophagus, colon) [70,73,76,77]. This lower metabolic resilience at sites of CD symptoms provides a metabolic explanation for CD tropism. Whether this is shaped by immune signals, parasite-derived molecules, microbiome metabolites, or all of these regulators in combination remains to be determined. Metabolic elasticity, a measure of the ability of metabolic pathways to respond to perturbations, decreases with age and varies between cell types [78]. A tissue-specific loss of metabolic elasticity with age may thus be associated with the progressive emergence of localized symptoms in CD over time.

Tissue purine depletion is observed during acute [72] and chronic infection in mouse models [73,77] and in humans [74], paralleled with increased levels of downstream molecules such as xanthine and urate [72]. These changes were correlated with indicators of disease severity in mice [73]. Conflicting results have been found in human studies: In one study, higher serum uric acid was found in patients with more severe disease [79], whereas no difference was observed between infected versus uninfected, asymptomatic versus symptomatic, or patients with or without cardiomyopathy in an older study [80]. Beyond CD, higher uric acid is associated with proportionally higher risk of cardiovascular disease mortality [81]. Given the anti-inflammatory role of adenosine, its role in promoting Th17 immune responses [82], and the pro-inflammatory role of uric acid under conditions where other inflammation-activating signals are present [83], these changes could also be exacerbating CD progression. Furthermore, uric acid impairs fatty acid metabolism [84] and thus could be contributing to disease-associated shifts in cardiac energy balance.

Specifically, changes in transcripts encoding for fatty acid metabolic enzymes in vitro [51] and in vivo [85], as well as changes in acylcarnitine abundance [70–73,76,77,86] (**Fig 1**), and in glucose clearance [86] and glycolytic intermediates [72], may reflect changes in cardiac energy balance between fatty acid oxidation and carbohydrate oxidation. Excessive reliance on fatty acid oxidation and a lack of myocardial metabolic flexibility are associated with worse outcomes in non-CD heart disease and may thus also be contributing to CD pathogenesis [87,88]. Indeed, treating mice with carnitine was associated with improved survival, in association with metabolic restoration and improved cardiac strain during acute *T*. *cruzi* infection [70]. In contrast, accumulation of glucose without adequate metabolic processing can lead to deleterious effects such as the formation of advanced glycation end products (AGEs, glycated host proteins), which can in turn promote oxidative damage [57,89,90]. Thus, the infection-associated increase in glucose import [57,58], as described above, could be one of the causes of the increased oxidative stress observed in CD [91].

Impaired function of complex III of the mitochondrial electron transport chain is observed in *T*. *cruzi-*infected mouse cardiomyocytes in vitro [92] and in the heart in acute and chronic *T*. *cruzi* infection in rodent models [93–98], though this differs from transcriptional analyses in the first 24 h of infection in human cardiomyocytes in vitro [99] and metabolomic analysis of human patients [85]. Not only would complex III impairment reduce ATP production [97] (though not in all studies; [94]) and thus affect cardiac muscle contractility, it also leads to electron leakage, causing the formation of damaging reactive oxygen species [98]. Increased mitochondrial reactive oxygen species production, increased myocardial hydrogen peroxide, and increased indicators of oxidative damage are observed in the hearts of mice acutely and chronically infected with *T*. *cruzi* [95,100,101].

Antiparasitic responses are fueled by specific metabolic shifts. M1 macrophages are primarily parasiticidal against *T*. *cruzi* and are important for acute-stage parasite control (see [102] for more details on the relative roles of M1 and M2 macrophages during *T*. *cruzi* infection). M1 macrophage activation with interferon gamma (IFNɣ) promotes macrophage glycolysis [103], and increased glycolysis is also observed in monocytes from CD patients [104]. Production of reactive oxygen and nitrogen species by *T*. *cruzi-*infected macrophages also necessitates glucose flux through the pentose phosphate pathway [105]. Thus, the hypoglycemia observed in some infection models could impair parasite clearance [106,107]. Impairing mitochondrial oxygen consumption also reduces macrophage nitric oxide production [108]. In endothelial cells, TNFɑ treatment can likewise increase glucose oxidation and TCA cycle flux, promoting pro-inflammatory gene expression [109]. Pro-inflammatory cytokine production by splenocytes requires fatty acid oxidation [110]. However, activating these metabolic pathways may also directly benefit the parasite. For example, pretreatment of cardiomyocytes with the immune stimulant lipopolysaccharide (LPS) increased parasite replication in a glucose import- or glycolysis-dependent manner [57]. Increased T cell glycolysis and oxidative phosphorylation are observed during acute *T*. *cruzi* infection in mouse models [111]. This may be helping parasite control, by fueling antiparasitic responses [104], but is also associated with mitochondrial damage that impairs immune responses [111]. In parallel, IFNɣ promotes fatty acid oxidation in endothelial cells [112], which can benefit *T*. *cruzi* proliferation [52]. Lastly, these reactive oxygen and nitrogen species, stimulated by pro-inflammatory cytokines, cause tissue damage and are direct causes of the mitochondrial impairment in CD [113].

## 5. Beyond cross-eukaryote interactions: Metabolic role of the mammalian microbiome in Chagas disease

While the mammalian microbiome has considerable metabolic effects (for instance, [114]) and drives the pathogenesis of many diseases, its metabolic role in CD remains relatively understudied. Early studies in germ-free mice showed worse disease outcomes than in conventional mice (for instance, [115]), however, with the caveat that such mice present with significant immune-maturation defects [116]. *T*. *cruzi* infection persistently perturbs the gut microbiome in mouse models [70,117,118] and in humans [119–121]. These changes were correlated with metabolic alterations, in particular in bile acids and fatty acids [70,117]. Bacterial metabolic genes encoding for key steps in fatty acid oxidation were increased by infection, with genes involved in fatty acid synthesis, short-chain fatty acid (SCFA) synthesis, and amino acid synthesis decreased by infection. The loss of anti-inflammatory SCFAs could be a direct driver of inflammation and especially gastrointestinal damage in CD [118]. Furthermore, loss of SCFA production could directly contribute to cardiac CD pathogenesis: SCFAs prevent mitochondrial damage and reactive oxygen species production and serve as an alternative cardiac fuel source [122]. However, it should be noted that these were metagenomic rather than metatranscriptomic or metaproteomic studies.

Given that these compositional changes persist following treatment [119], the microbiome may also be responsible for maintaining some of the metabolic changes that persist after *T*. *cruzi* clearance, such as persistent purine depletion [73]. Indeed, purine biosynthetic genes are at lower levels in the microbiome of infected mice [118]. A causal link between gut bacteria, plasma levels of purines and uric acid, and non-CD cardiovascular disease severity has recently been demonstrated [123].

## 6. External influences: Role of the diet

**Fig 2:**
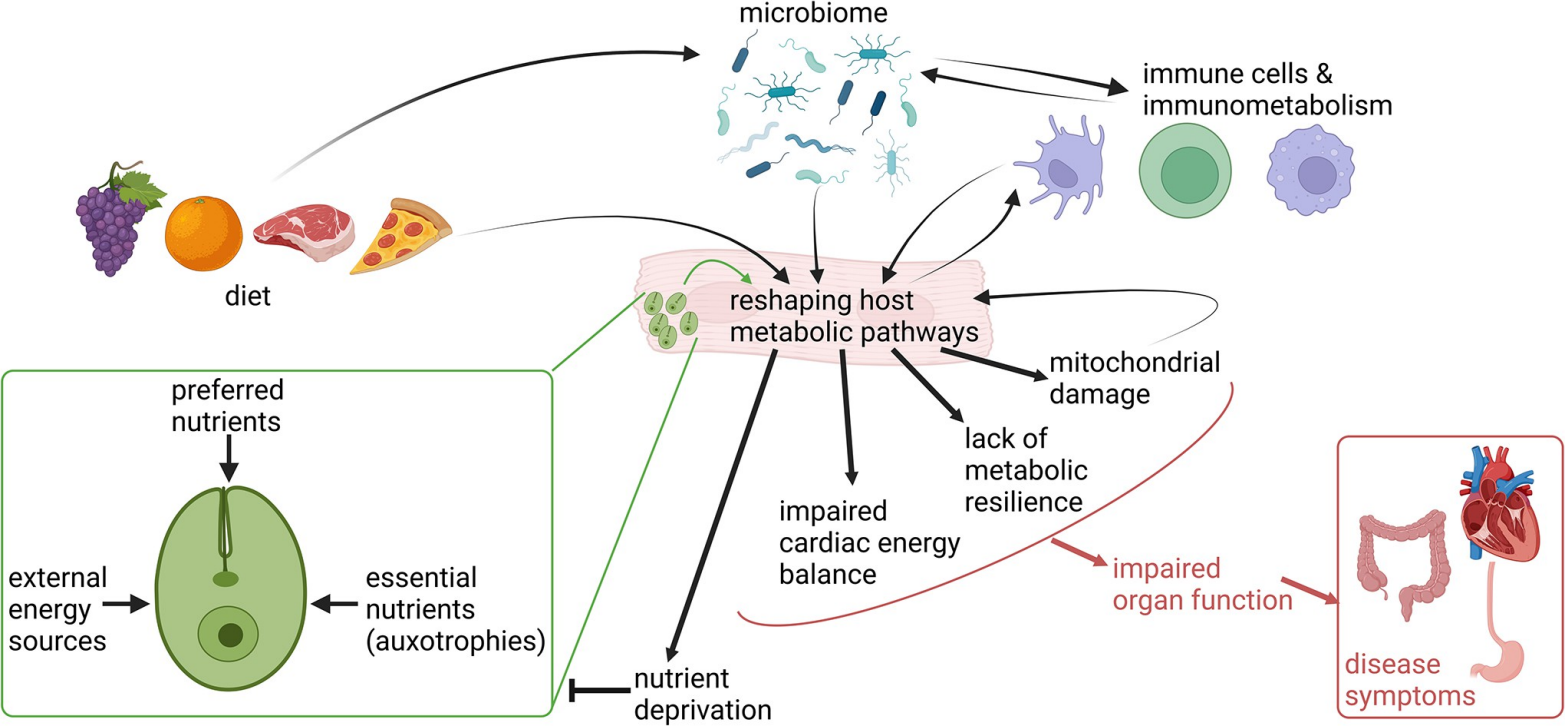
Conceptual overview of metabolic interactions between *T*. *cruzi*, the microbiome, and the mammalian host. Curved arrows indicate interactions. Diet, *T*. *cruzi*, the microbiome and immune responses all reshape host metabolic pathways. Some of these changes can impair parasite growth or promote antiparasitic immune responses, while other changes are maladaptive and lead to impaired organ function and disease symptoms. Figure created with BioRender.com.

Beyond host and parasite genetics, human behavior also impacts disease progression (**Fig 2**). The best studied behavioral determinant of CD progression is diet and its effects on metabolism, intersecting with parasite invasion, parasite proliferation, and antiparasitic immune responses. Severe malnutrition led to earlier mortality in experimental models of acute *T*. *cruzi* infection, likely through depressed immune responses [124,125]. These results concur with findings in the context of *Plasmodium* infection, where chronic malnutrition was correlated with increased disease severity [126]. Protein deficiency also led to earlier and/or higher parasitemia in two acute infection models [127,128], whereas a third study only observed elevated parasitemia in mice receiving a high protein diet [129]. The postulated mechanism for worsened outcomes in low-protein settings is reduced inflammatory responses, impairing parasite clearance [128,130], and higher levels of the vasoconstrictor endothelin-1 [128].

Several studies analyzed the impact of shifting lipid homeostasis through a high fat diet (**Table 1**). In the acute stage, worse parasitemia was observed in mice fed a high-fat diet [131–134], in association with higher cardiac inflammation [131,133], though this contrasts with [135,136]. The functional impact during chronic infection was variable depending on the ventricle [137]. In contrast, restricting fatty acids 10-fold by diet weight reduced parasitemia and improved survival [138]. This may be linked to the parasite’s reliance on scavenging host lipids [63]. Obesity increased the severity of acute experimental CD in mouse models, with higher mortality, higher parasite burden, higher oxidative stress, and higher pro-inflammatory cytokines [139]. This contrasts with findings in the context of malaria infection, where a high-fat diet impaired *Plasmodium* liver infection [140]. One caveat with these studies is that many of the high-fat diets are also higher caloric (for instance, [131,136]); thus, the observed effects may reflect caloric differences rather than purely fat-driven effects.

**Table 1:** Effect of high fat diet on experimental CD outcomes.

Parasite strain (DTU)	Mouse strain	Mouse sex	Time point	Diet	Effect on cardiac parasite burden	Effect on extracardiac parasite burden	Effect on parasitemia	Effect on inflammation	Proposed mechanism	Reference
Brazil (TcI)	CD-1	M	Acute (to day 30)	60 kcal% fat (reference is 10% fat)	Reduced in high-fat diet	Increased in adipose tissue in high-fat diet	Reduced in high-fat diet	Reduced	Sequestration of parasites in adipose tissue	[135]
Berenice-78 (TcII)	Swiss	F	Acute (to day 30)	452 kcal% and 20% fat (reference is 332 kcal% and 4% fat)	Not assessed	Not assessed	Reduced in high-fat diet	Not assessed	Sequestration of parasites in adipose tissue	[136]
VL-10 (TcII)	C57BL/6	F	Acute (to day 30)	60% of calories come from fat (reference is 10%)	Not assessed	Not assessed	Increased by high-fat diet	Higher cardiac TNFɑ, adipose TNFɑ, adipose IFNɣ with high-fat diet; no differences in cardiac IFNɣ	Effects of cholesterol on parasite invasion and proliferation	[131]
Brazil (TcI)	CD-1	M	Acute (to day 30)	60 kcal% fat (reference is 10% fat)	Not assessed	Increased liver parasite burden	Not assessed	Reduced inflammation in the liver under high-fat diet, reduced TNFɑ	Less antioxidants	[156]
VL-10 (TcII)	C57BL/6	M	Acute and chronic	60% of calories come from fat (reference is 10%)	Not assessed	Not assessed	Increased by high-fat diet	Higher plasma IL10 and TNFɑ at 12 wk; no difference in cardiac inflammatory infiltrate; less liver inflammatory infiltrate		[134]
Y (TcII)	C57BL/6	M and F	Acute	Fish oil or corn oil at approximately 5%–6% of daily energy intake	Reduced by fish oil only at day 12		Significantly higher at day 7 only in the fish oil group	Reduced nitric oxide in the fish oil groups only at time points matching observations on parasite levels; reduced PGE2 production by splenocytes at day 12; increased MCP1		[132]
Colombian (TcI)	C57BL/6	M	Acute to early chronic (to day 60)	17.8% olive oil added to regular diet or 16.8% lard added to regular diet (no significant differences in calorie intake)	Increased by lard diet at day 30	Increased liver parasite burden by lard diet at day 30	None	Heart CCL2 increased by lard and adipose CCL2 decreased; no effect on cellular infiltration	Limited mortality overall	[157]
Brazil (TcI)	CD-1	Not specified	Acute and early chronic (to day 70)	60 kcal% fat (reference is 10% fat)	Not assessed	Not assessed	Later peak parasitemia, higher parasitemia in high-fat diet	Higher IFNɣ and TNFɑ in the hearts of mice under high-fat diet (day 30); no differences in plasma IFNɣ and TNFɑ at day 70	Lower mortality in high-fat diet; enhanced glucose tolerance in high-fat diet	[133]
Brazil (TcI)	CD-1	M	Chronic (day 100–150)	60 kcal% fat (reference is 10% fat)	Not assessed	Not assessed	Not relevant (chronic stage)	High-fat reduced inflammatory infiltration in right ventricle only (not in left ventricle), at 120 days postinfection; increased cardiac TNFɑ and IFNɣ at 120 days postinfection but decreased at 160	High-fat diet improved left ventricle internal diameter but altered right ventricle internal diameter; worse cardiac hypertrophy with high-fat diet in left ventricle at day 120 and in right ventricle at day 160	[137]
Berenice-78 (TcII)	Swiss	F	Chronic (day 90)	452 kcal% and 20% fat (reference is 332 kcal% and 4% fat)	Not assessed	Not assessed	Not relevant (chronic stage)	No significant impact on serum TNFɑ or cardiac inflammatory infiltrate	Lipolysis in infected animals	[136]

Studies in humans show more conflicting results. In two studies, CD patients had lower body mass index (BMI) than controls [141–143], whereas a study of indeterminate state CD and symptomatic patients had higher BMI than controls [144,145], in association with increased blood triglycerides [145]. Proportional associations between BMI and disease severity have also been reported [146]. Patients with anti-*T*. *cruzi* antibodies were less likely to be PCR positive if they were overweight or obese, suggesting differential parasite dynamics and, possibly, sequestration based on patient adipose tissue [147], mirroring findings in mice [61,136]. Confounders include gastrointestinal discomfort in CD patients, which could have led to the dietary alterations, study sites, and socioeconomic factors [141,142]. Whether effects are direct, or via gut microbiota modulation, is also unclear.

Other nutrients can also alter outcomes of infection. Long-term vitamin C supplementation increased cardiac damage at the chronic stage [148], while acute-stage treatment reduced parasitemia, cardiac parasite burden, and cardiac inflammation [149]. Vitamin A, B1, B5, and B6 deficiency increases parasitemia and cardiac damage in a rat model of infection [150–153]. In contrast, little effect was observed for vitamin B2 deficiency [154]. Lysine supplementation reduces parasitemia and improves survival [155]. Overall, dietary effects over an infected individual’s life span are thus likely to strongly impact disease progression but require further study, which may be challenging due to the need for large cohorts and long-term follow-up.

## 7. Translational applications: Chagas disease treatment

### 7.1 Relationship between metabolism and parasitological treatment failure

Treatment failure can be divided into parasitological treatment failure, where residual parasites persist after antiparasitic treatment, and clinical treatment failure, when parasite clearance is achieved but patient symptoms do not resolve. The current antiparasitic drugs nifurtimox and benznidazole require activation by *T*. *cruzi* type I nitroreductase for activity. The endogenous role of this enzyme is still unclear, but it may be involved in electron transfer from reducing equivalents like NADH, with a postulated critical role in epimastigote to trypomastigote differentiation and infectiveness [158]. Expression levels and activity of this nitroreductase control drug sensitivity [159], though other factors are also involved as natural isolates with variable benznidazole sensitivity did not have a clear correlation with nitroreductase sequence [160]. Metabolic context is also a critical determinant of drug efficacy. Parasitological treatment failure with benznidazole has been linked to parasite dormancy [161], but the factors regulating dormancy in *T*. *cruzi* are still unknown. Benznidazole treatment directly leads to DNA damage that may promote dormancy, but preexisting dormancy is also observed even in the absence of benznidazole treatment [161,162]. Interestingly, in the related parasite *Leishmania donovani*, dormancy has been tied to purine depletion [163], so that the lower purine levels observed during *T*. *cruzi* infection could be contributing to this phenomenon [72–74,77]. Glutamine metabolism also modulates the efficacy of azole treatments in eliminating intracellular *T*. *cruzi* amastigotes, independently of parasite growth rate [164]. The colon had lower steady-state glutamine than other tissues [70]. Additionally, the intestine is a major site of glutamine absorption and metabolism [165]. Thus, variable availability of glutamine in the colon may contribute to parasite persistence at that site following azole treatment [166].

### 7.2 Treatments affecting *T*. *cruzi* metabolism

Targeting metabolic pathways that are unique to *T*. *cruzi* metabolism, which use divergent enzymes compared to the homologous mammalian enzyme, or that are essential only in *T*. *cruzi* and not the mammalian host, is a common strategy for drug development. Genome-scale metabolic models provide candidates, such as jointly targeting parasite glutamate metabolism and the citric acid cycle, or glutamate metabolism and oxidative phosphorylation [167]. Indeed, the development of azoles for CD treatment relied on differential sterol profiles between host and parasite [168]. Selectivity is achievable even with enzymes shared between *T*. *cruzi* and the host, as demonstrated by GNF7686, which inhibits mitochondrial complex III of the electron transport chain in *T*. *cruzi* only [169].

### 7.3 Immune modulation through metabolism to improve Chagas disease symptoms

While the goal of current CD treatments is to clear the parasite, this is insufficient to fully restore infection-associated metabolic alterations [73,75,170,171]. Metabolic modulators are uniquely poised to address these changes, renormalize metabolism, and improve disease symptoms (**Table 2**). Large-scale immunomodulation through a therapeutic vaccine provided superior metabolic restoration compared to antiparasitic treatment with benznidazole alone, in parallel with improved IFNɣ levels [73]. Aspirin inhibits inflammatory prostaglandins while also increasing the levels of the anti-inflammatory lipid mediator 15-epi-lipoxin A4. This improved mean arterial pressure and decreased heart rate and hypertension in infected animals. However, aspirin reduced cardiac parasite burden, suggesting that these effects could be due both to direct parasite clearance, as well as metabolic and immune modulation [172].

**Table 2:** Representative metabolism-modulating strategies tested in CD mouse models.

Parasite strain (DTU)	Mouse strain	Mouse sex	Time point of the treatment	Treatment	Effect on cardiac parasite burden	Effect on parasitemia	Effect on electrocardiographic and echocardiographic parameters	Effect on cardiac fibrosis	Effect on inflammation	Proposed mechanism	Reference
Colombian (TcI)	BALB/c	M or F	60–90 days postinfection	Metformin (500 mg/kg)	Unchanged		Improved ECG, improved cardiac pumping			Reduced oxidative stress	[180]
Brazil (TcI)	CD-1	Not specified	Day -20 to day 71 postinfection	Metformin (50 mg/kg)		Reduced			Unchanged	Reduced oxidative stress	[133]
Colombian (TcI)	BALB/c	M or F	60–90 days postinfection	Resveratrol (15 mg/kg)	Reduced		Improved ECG, improved cardiac pumping			Reduced oxidative stress	[180]
Y (TcII)	BALB/c	M	2–32 days postinfection	Aspirin (25 mg/kg)	Reduced	Reduced	Reduced heart rate, mean arterial pressure	Reduced	Increased eosinophils, reduced neutrophils; increased nitrate	Anti-inflammatory	[172]
Y (TcII)	BALB/c	Not specified	Not specified—study ended 21 days postinfection	L-arginine in drinking water (3.75 mg/ml)	Decreased	Decreased	Improved response to cardiac stress		Increased plasma nitrites	Compensating for infection-induced arginine deficiency, leading to better parasite killing	[173]
Sylvio X10 (TcI)	C57BL/6	Not specified	45–66 days postinfection	SRT1720, 1 mg/mouse	Unchanged		Multiple parameters improved	Unchanged	Reduced	Reduced oxidative stress and inflammation	[174]
CL Brener + luciferase (TcVI)	C3H/HeJ	M	7–17 days postinfection	Carnitine (in drinking water at 100 mg/kg per day)	Unchanged	Unchanged	Assessed indirectly: improved levels of BNP as a marker of cardiac strain	Unchanged	Unchanged	Improved cardiac metabolism leading to improved cardiac function	[70]
Colombian (TcI)	C57BL/6	F	120–150 days postinfection	Pentoxifylline (20 mg/kg)	Unchanged	Unchanged	Improved	Reduced	Reduced	Immunomodulation	[177]
Colombian (TcI)	C57BL/6	F	120–150 days postinfection	Pentoxifylline (20 mg/kg)	Unchanged		Improved	Reduced	Reduced	Immunomodulation	[178]

Similarly, L-arginine is metabolized by the inducible nitric oxide synthase pathway (iNOS), which produces nitric oxide responsible for killing the parasite. Arginine is reduced during acute infection. L-arginine treatment decreased parasitemia and reduced cardiac hypertrophy [173]. Ameliorating cardiac inflammation and oxidative damage through treatment with a SIRT1 agonist improved cardiac function, without altering cardiac parasite burden or cardiac fibrosis [174]. Antioxidant treatments are also being tested in patients, with some promise in late-stage disease, though most studies did not assess functional improvement (see [175] for a systematic review).

Pentoxifylline is a phosphodiesterase inhibitor that reduces proinflammatory cytokines through the manipulation of cyclic adenosine monophosphate levels (cAMP). Treating chronically infected mice with benznidazole, pentoxifylline, or the combination of the two reduced TNFα signaling. Pentoxifylline and benznidazole also reduced cardiac fibrosis, cardiac hypertrophy, and cardiac electrical abnormalities [176–178]. Overall, modulating immune responses through metabolism is a promising strategy for CD treatment, but studies in CD patients are needed.

### 7.4 Beyond immunity: Alternative metabolic restoration strategies for Chagas disease

Treatment with carnitine during acute experimental *T*. *cruzi* infection builds on findings of infection-induced changes in acylcarnitines [70–73,76,77,86]. Carnitine treatment prevented acute mortality, improved cardiac strain, and reset host cardiovascular metabolism, mitigating infection-induced metabolic disruptions in the plasma and heart, with no effect on immune responses. This was evident in the distinct metabolic profiles of vehicle-treated animals compared to uninfected or benznidazole-treated animals, with carnitine-treated infected animals showing a reduced difference to uninfected samples. However, carnitine treatment had a comparatively minor impact on the overall metabolite profiles of the esophagus and large intestine and did not restore metabolism in these tissues [70]. The specific mechanism of action of carnitine is still under investigation, but the fact that carnitine is at the nexus of fatty acid and carbohydrate oxidation [179] suggests the possibility that synergistic effects or combination treatments that target multiple metabolic pathways may be the best approach. Such multifactorial mechanism of action may also underlie the protective effects of metformin in CD [133,180]. Metformin is in clinical use for diabetes; it inhibits gluconeogenesis but also has antioxidant and immunomodulatory properties, alters protein synthesis, and promotes lipolysis and fatty acid oxidation [181]. In vitro, treatment with the experimental compound named S205 (structurally undefined in the source manuscript) provided superior overall proteome restoration and pyruvate and lactate levels [171]. These results indicate the potential of metabolic restoration as a treatment strategy for CD. Restoring purine metabolism, for example, via allopurinol, may be an interesting avenue to revisit [182], given the intersection between purine metabolism and immunity [82], and the fact that nucleotides and nucleosides are strikingly harder to renormalize with standard antiparasitic treatment [73].

## 8. Challenges and opportunities

New and newly implemented technologies such as single-cell mass spectrometry, spatial metabolomics, and microbiome metagenomics [70,118,183] are increasingly providing insight into the small molecules and metabolic pathways shaping host-*T*. *cruzi-*microbiome-environment interactions (**Box 1**). Given that the *T*. *cruzi* lipidome differed between amastigotes isolated from different host cell lines in vitro [63], there is considerable potential for reshaping of the *T*. *cruzi* lipidome and metabolome depending on host cell type and tissue context in vivo. New techniques to analyze low-frequency cells [184,185] will therefore be critical to understand parasite metabolic shifts within the context of infected tissues. Reduction in costs and greater accessibility of these techniques has increased their implementation in the context of CD.

**Box 1. Challenges, gaps, and new techniques**

- Challenges
  1. Complexity of host-*T*. *cruzi-*microbiome-environment interactions and their interdependence
  2. Interdependence of metabolic pathways
  3. Effect of spatial context
  4. Bystander effects
  5. Dynamicity of metabolic changes, whereas any data acquisition is by its very nature a specific single moment in time
  6. Discrepancies between studies: usage of different parasite strains, different mouse strains, and different time points
  7. *T*. *cruzi* genetic diversity
  8. Interperson and temporal variability, compounded by the slow progression of CD
  9. Hard-to-access human samples
  10. Transcriptome-level and protein-level analyses may not reflect metabolic flux
- Gaps in the field
  1. Impact of other aspects of diet and behavior, beyond high-fat diet
  2. Life course/life history effects, including infections with other pathogens
  3. Coinfections, superinfections, and how they alter signaling pathways
  4. Parasite gene expression in situ, inside tissues, and local parasite metabolic alterations
  5. Concentrations of key nutrients inside cells and their availability to the parasite
  6. Effect of microenvironment, tissue, and inflammatory context on nutrient availability and parasite metabolic decisions
  7. Mechanisms and consequences of bystander effects
  8. Determinants of tissue metabolic resilience or lack thereof
  9. Determinants of persistent metabolic changes after antiparasitic treatment
  10. Signals and processes critical for *T*. *cruzi* development or growth inhibition in triatomines
  11. Lack of information on triatomine salivary, urinary and fecal metabolites and their role in initiating infection
  12. In vivo metabolic flux analyses
- New techniques
  1. Spatially aware single-cell analyses
  2. Multiorgan and cross-organ approaches like chemical cartography
  3. New data analysis techniques, beyond classical correlations with their high false positive rates
  4. Gentler ways to purify cells that don’t cause artefactual metabolic changes
  5. Cost reduction enabling greater implementation of flux analysis

However, cross-study comparability remains limited by the divergence in mouse strains, parasite strains, and time points studied between laboratories. This is particularly concerning given the broad genetic diversity within *T*. *cruzi*, in association with divergent disease symptoms [15,186]. However, this also represents an opportunity for systematic studies to unravel the intersection of parasite genetics, host genetics, and metabolism with CD pathogenesis. Coinfections and superinfections should also be considered, particularly in light of recent findings that intrahost parasite strain diversity is correlated with parasitemia control and slower deterioration of ECG parameters [187].

Given that only a minority of *T*. *cruzi-*infected individuals progress to severe CD [14], understanding the impact of life history (including infections with other pathogens) and cumulative behavioral effects beyond diet has the potential to provide more personalized estimates of disease progression and patient outcomes. However, studies of metabolism in humans are challenging due to the strong susceptibility of metabolism to postmortem effects, its spatial and temporal dynamicity, and the interindividual variability compounded by the long-term nature of CD.

A further challenge is the complexity of these interactions and their interdependence: immunity shapes metabolism, metabolism shapes immunity, both are influenced by the microbiome, and all can be affected by *T*. *cruzi* and by patient behavior. These linkages and cross-system feedbacks make determination of causality challenging. A new systems perspective is thus necessary, which considers the cumulative effect of small codependent interactions, perhaps conceptually extending from a framework similar to polygenic risk scores in genetics (for instance, [188,189]). This will need new data analysis frameworks, supported by increased implementation of time-course analyses rather than single-time point studies. A further, underappreciated interdependence is between metabolic pathways themselves. Indeed, carbohydrate oxidation and lipid oxidation, for example, are cross-regulated [179]. Inter-organ communication and variability also need to be considered, as do fine-scale within organ and cell-to-cell variability and effects. Indeed, single-cell metabolomics has revealed bystander effects of infection, where uninfected but infection-adjacent cells also show metabolic shifts [57,183]. Bystander cells have been ignored in traditional antiparasitic drug development but may prove valuable targets for metabolism-modulating therapeutics. The mechanisms establishing and maintaining bystander effects and persistent metabolic changes following antiparasitic treatment remain to be determined. Cascading bystander effects and immune-mediated metabolic regulation will also be critical to understand how such low and localized parasite burden during chronic infection can nevertheless lead to metabolic changes on a macroscopic level. Overall, expanding our understanding of these interactions will lead to new ways to monitor and interrupt CD progression, focused on disease mechanisms.
